# Use of Azacitidine or Decitabine for the Up-Front Setting in Acute Myeloid Leukaemia: A Systematic Review and Meta-Analysis

**DOI:** 10.3390/cancers13225677

**Published:** 2021-11-12

**Authors:** Miriam Saiz-Rodríguez, Jorge Labrador, Beatriz Cuevas, David Martínez-Cuadrón, Verónica Campuzano, Raquel Alcaraz, Isabel Cano, Miguel A. Sanz, Pau Montesinos

**Affiliations:** 1 Research Unit, Fundación Burgos por la Investigación de la Salud (FBIS), Hospital Universitario de Burgos, 09006 Burgos, Spain; ralcaraz@hubu.es; 2 Department of Health Sciences, University of Burgos, 09001 Burgos, Spain; 3 Hematology Department, Hospital Universitario de Burgos, 09006 Burgos, Spain; bcuevas@saludcastillayleon.es (B.C.); vcampuzano@saludcastillayleon.es (V.C.); 4 Hematology Department, Hospital Universitari i Politècnic La Fe, 46026 Valencia, Spain; martinez_davcua@gva.es (D.M.-C.); cano_isafer@gva.es (I.C.); Miguel.Sanz@uv.es (M.A.S.); montesinos_pau@gva.es (P.M.)

**Keywords:** azacitidine, decitabine, meta-analysis, acute myeloid leukaemia, elderly

## Abstract

**Simple Summary:**

Azacitidine and decitabine have been increasingly used for the treatment of acute myeloid leukaemia in older patients. The choice between azacitidine and decitabine depends mostly on the experience and preference of the attending physician, since they have not been compared directly in a randomised clinical trial. In this study, we identified the best treatment regimen for each drug and compare the efficacy of decitabine and azacitidine monotherapy in newly diagnosed acute myeloid leukaemia. We found no significant differences regarding 1-year mortality and overall survival for azacitidine and decitabine (roughly 9 months). Moreover, there were no significant differences in the efficacy of 5-day versus 10-day schedules of decitabine. However, patients treated with the shortened 5-day azacitidine scheme showed worsened outcomes compared to the standard 7-day regimen. Hopefully, our results might be helpful for the design of azacitidine/decitabine-based combination schedules to be tested in future trials.

**Abstract:**

Irruption of decitabine and azacitidine has led to profound changes in the upfront management of older acute myeloid leukaemia (AML). However, they have not been directly compared in a randomised clinical trial. In addition, there are no studies comparing the optimal treatment schedule of each drug in AML. A systematic review and meta-analysis on the efficacy of decitabine and azacitidine monotherapy in newly diagnosed AML was conducted. Randomised controlled trials and retrospective studies were included. A total of 2743 patients from 23 cohorts were analysed (10 cohorts of azacitidine and 13 of decitabine). Similar response rates were observed for azacitidine (38%, 95% CI: 30–47%) compared to decitabine (40%, 95% CI: 32–48%) (*p* = 0.825). Overall survival (OS) between azacitidine (10.04 months, 95% CI: 8.36–11.72) and decitabine (8.79 months, 95% CI: 7.62–9.96) was also similar (*p* = 0.386). Patients treated with azacitidine showed a lower median OS when azacitidine was administered for 5 days (6.28 months, 95% CI: 4.23–8.32) compared to the standard 7-day schedule (10.83 months, 95% CI: 9.07–12.59, *p* = 0.002). Among patients treated with decitabine, response rates and OS were not significantly different between 5-day and 10-day decitabine regimens. Despite heterogeneity between studies, we found no differences in response rates and OS in AML patients treated with azacitidine or decitabine.

## 1. Introduction

Acute myeloid leukaemia (AML) is a heterogeneous haematological malignant disease characterised by clonal abnormal proliferation of immature haematopoietic progenitor cells of the myeloid lineage [1]. AML is commonly acknowledged as a disease of older adults, the median age being 68 years old [2,3]. In fact, the incidence of AML in Europe is 3.7 per 100,000 among the general population but increases to 13.7 per 100,000 among people aged 65 or older [4].

Although advances in AML treatment have improved the overall outcome of patients, the prognosis for older patients remains poor [5,6], with roughly 70% of patients over the age of 65 dying within the first year of diagnosis [7]. The toxicity and the high treatment-related mortality associated with intensive chemotherapy, comorbidities and poorer performance status limit its use in the elderly [8]. In addition, older AML patients are more likely to have secondary AML, adverse cytogenetics or the multidrug resistance phenotype, which raises the issue of whether they should receive palliative care [9,10,11,12]. Acceptable options for older patients include low-dose Ara-C and hypomethylating agents (HMAs), which increase the proportion of patients receiving active therapy [6]. Epigenetic changes in AML, especially in aberrant DNA methylation, play a role in the regulation and expression of tumour suppressor genes and oncogenes [13]. As epigenetic changes, these aberrant modifications are reversible, making them potential therapeutic targets. Azacitidine and decitabine are pyrimidine nucleoside analogues that inhibit DNA methyltransferase, impairing DNA methylation. The clinical and biological efficacy of HMAs has been proven in numerous in vitro/in vivo studies and clinical trials [13,14]. Therefore, HMAs have been increasingly used for older AML patients. Their different mechanism of action associated with a manageable toxicity profile, low incidence of mortality and administration in the outpatient setting make them ideal agents for use in older patients [15].

Both azacitidine and decitabine are well tolerated, and they have shown improved overall survival compared with the best supportive care or low-dose Ara-C [16,17]. Despite the widespread use of these agents, there is no consensus regarding their comparative efficacy and clinical benefit, with notable between-study variability [1].

At present, the effectiveness of decitabine and azacitidine has not been compared directly in a randomised clinical trial. Indeed, the choice of HMA depends mostly on the experience and preference of the attending physician.

In addition, there are limited studies comparing the optimal treatment schedule of each drug in AML. Higher response rates have been reported with a 10-day schedule of decitabine [18], although these results have not been validated [19], leading to the need for randomised clinical trials comparing it with standard 5-day schedule. Similarly, studies have reported the efficacy of azacitidine with doses different from the standard 7-day schedule [20,21], but there are no randomised trials.

The scarcity of well-controlled studies performed in older AML patients accentuates the usefulness of well-conducted meta-analyses. In this systematic review and meta-analysis, the aim was to analyse and compare the efficacy of either decitabine or azacitidine monotherapy in newly diagnosed AML patients in order to shed light on the management of an already difficult-to-treat population. Moreover, this study also aimed to compare the efficacy and survival of a standard dose of azacitidine and decitabine versus other dose regimens for each drug.

## 2. Materials and Methods

The protocol for this study was developed a priori and registered in PROSPERO (ID CRD42020181405). This systematic review was conducted according to the Preferred Reporting Items for Systematic Reviews and Meta-Analyses (PRISMA) statements [22].

### 2.1. Eligibility Criteria

The study included randomised controlled trials and retrospective studies that recruited adults with newly diagnosed AML who were treated with either azacitidine or decitabine monotherapy. Trials were selected if performed on patients not eligible for intensive chemotherapy. The meta-analysis only included data from the azacitidine or decitabine monotherapy arms; data from experimental arms were excluded from the analysis.

Studies were included if at least one of the following outcomes was reported: mortality, overall survival (OS), complete remission (CR), complete remission with incomplete haematological recovery (CRi) and partial response (PR).

### 2.2. Data Sources and Search Strategies

The literature search included the following databases: MEDLINE, EU Clinical Trials Register and ClinicalTrials.gov. We additionally reviewed the reference lists of the most relevant clinical studies and review articles in order to be as comprehensive as possible. The complete search strategy is provided in Supplementary Table S1.

### 2.3. Study Selection

Two independent reviewers (M.S.-R. and J.L.) independently screened all the titles and abstracts and evaluated each article based on the eligibility criteria. Disagreements were resolved by consensus and in concordance with a third reviewer (P.M.), when necessary. Studies that fulfilled the following criteria were included: (1) newly diagnosed AML patients’ cohorts in which azacitidine or decitabine was prescribed upfront and (2) studies reporting efficacy and outcome variables.

### 2.4. Data Extraction and List of Variables Included

Two reviewers (M.S.-R. and J.L.) independently extracted data using standardised forms created in Microsoft Excel 2010. These forms contained the following information:

- Study (first author, year)
- Study design: phase I, II or III clinical trial, retrospective, prospective
- Intervention (dose, schedule): azacitidine or decitabine
- Comparison (description): best supportive care, low doses of cytarabine
- Patients (*N*)
- Age (years): median and range
- Male (%)
- ECOG 0/1, 2, ≥3 (%): Eastern Cooperative Oncology Group (ECOG) scale
- AML type (%): de novo or secondary
- Cytogenetics (%): favourable, intermediate, adverse
- BM blasts (median %): bone marrow blasts
- WBC count (10^9^/L median): white blood cell count
- CR (%): complete remission
- CCR (%): composite complete remission rate (CR + CRi)
- PR (%): partial response
- ORR (%): overall response rate (CR + Cri + PR)
- Median OS (months): overall survival
- Early mortality (first 30 and 60 days since randomisation) (%)
- Mortality (%)

### 2.5. Definitions

The definition of CR was consistently applied to all studies as the bone marrow blast count <5%, absence of circulating blasts and blasts with Auer rods; the absence of extramedullary disease; absolute neutrophil count >1.0 × 10^9^/L; and platelet count >100 × 10^9^/L [23]. CR/CRi included patients with a CR and patients who met all CR criteria but only one of these: absolute neutrophil count >1.0 × 10^9^/L or platelet count >100 × 10^9^/L, which is considered CRi. The OS was included when counting all deaths regardless of the cause and whether the patient received subsequent therapy. When the OS was not explicitly reported in the studies, it was estimated from Kaplan–Meier curves, if available. Early mortality data registered comprised 30-day mortality and 60-day mortality (proportion of patients who died within the first 30/60 days of treatment).

### 2.6. Methodologic Quality and Risk of Bias

We used Cochrane Collaboration’s tool for bias assessment [24], which includes the following items: random sequence generation, allocation concealment, selective reporting, blinding (of participants and personnel and of outcome assessment), incomplete outcome data and other sources of bias. This tool allows the classification of the risk of bias as low, unclear or high. Two reviewers (M.S.-R. and J.L.) independently assessed the risk of bias for each study. Disagreements were solved by consensus or by the intervention of a third reviewer (P.M.).

### 2.7. Statistical Analysis

Analyses were performed using Epidat v.3.1 software or STATA v.15 software, where applicable. For the estimation of variability, the following measurements were used: inter-study variance, intra-study variance and inter-study coefficient of variation. The I^2^ coefficient, representing the proportion of total variance explained by the inter-study variance, was used as the main estimator of variability, with I^2^ > 50% suggesting substantial heterogeneity. For evaluation of the ORR, mortality and OS, a random effects meta-analysis was performed according to the studies’ variability in outcomes for all endpoints. The results were summarised using a point estimate and the 95% confidence interval (CI). For analysis of the OS, the median and CI were transformed into the mean and SD, as described by Hozo et al. [25]. All analyses were based on the intention-to-treat (ITT) principle.

## 3. Results

### 3.1. Study Selection

**Figure 1:**
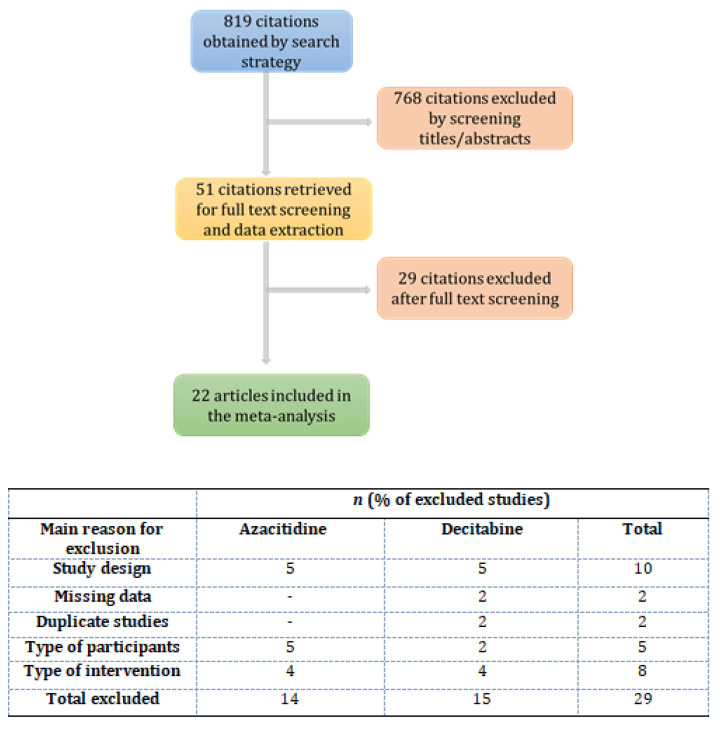
Flowchart of study selection procedure and the main reasons for article exclusion.

The search strategy retrieved 819 citations before removal of duplicates. The PRISMA flowchart of the selection procedure and the main reasons for exclusion are detailed in Figure 1.

### 3.2. Study Characteristics

A total of 2743 patients, from 23 cohorts (10 of azacitidine and 13 of decitabine), from 22 studies, were analysed. The characteristics of the included studies are depicted in Table 1, and the efficacy outcomes are shown in Table 2.

### 3.3. Outcomes

**Figure 2:**
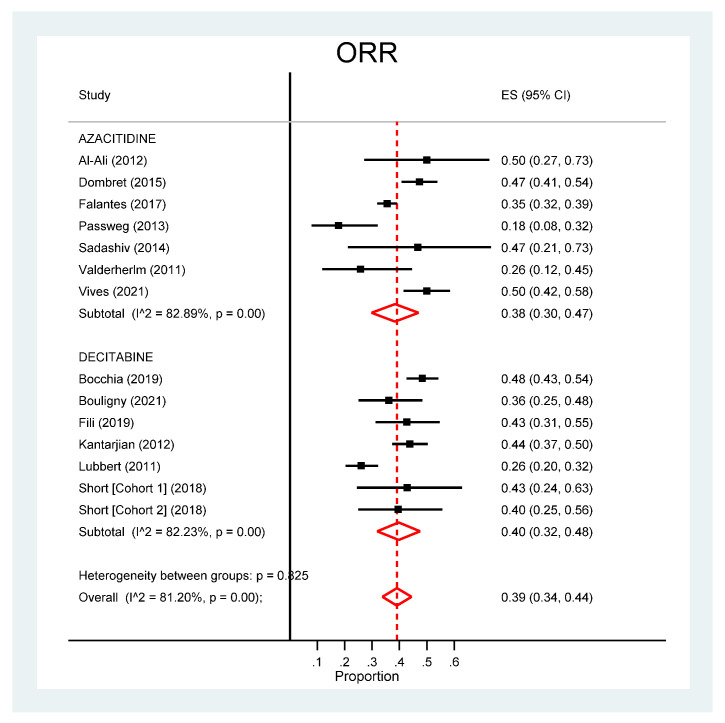
Pooled analysis of the overall response rate (ORR).

A significantly higher CR rate was observed for decitabine (25%, 95% CI: 20–30%) compared to azacitidine (16%, 95% CI: 12–19%) (*p* = 0.005). However, these differences disappeared when analysing the CCR, which was 27% (95% CI: 17–38%) for decitabine and 23% (95% CI: 14–32%) for azacitidine (*p* = 0.542). A lower ORR was observed for azacitidine (38%, 95% CI: 30–47%) compared to decitabine (40%, 95% CI: 32–48%) (*p* = 0.825), both with high I^2^ coefficients (>82%) (Figure 2).

The mortality rate at 30 days was not statistically different between azacitidine (6%, 95% CI: 4–9%) and decitabine (7%, 95% CI: 5–9%) (*p* = 0.724). However, a trend towards a lower mortality rate at 60 days was noticed for azacitidine (15%, 95% CI 11–19%) compared to decitabine (20%, 95% CI 16–23) (*p* = 0.107).

**Figure 3:**
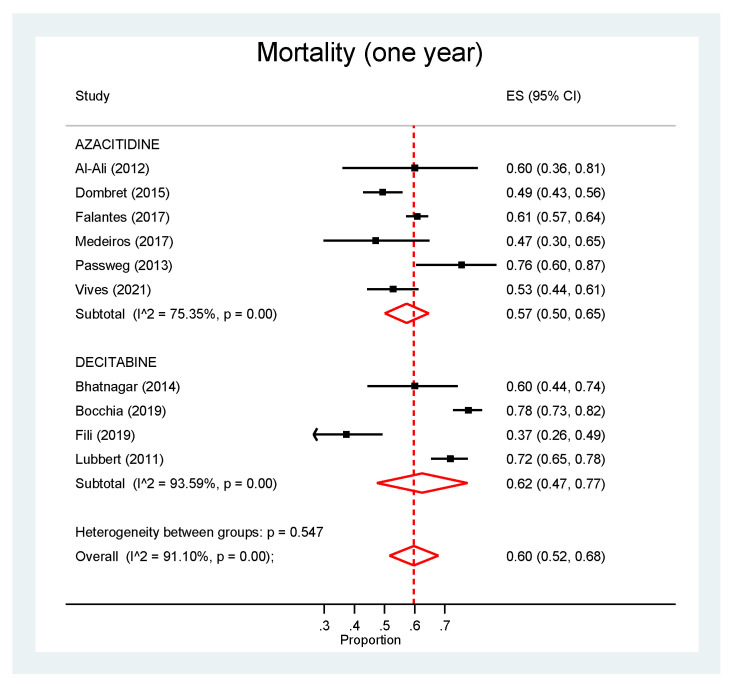
Pooled analysis of 1-year mortality.

Regarding 1-year mortality (Figure 3), we found no significant difference between azacitidine (57%, 95% CI: 50–65%) and decitabine (62%, 95% CI: 47–77%) (*p* = 0.547). However, again high heterogeneity was observed between studies, being over 75% for both agents.

**Figure 4:**
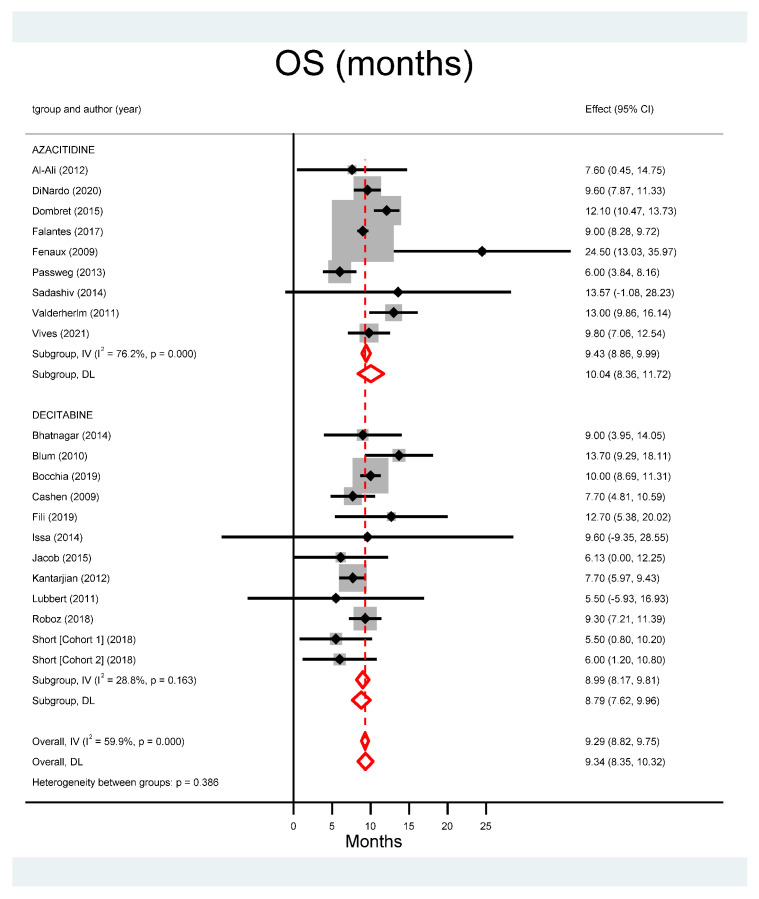
Pooled analysis of overall survival (OS).

Figure 4 depicts the median OS of all studies, being 10.04 months for azacitidine (95% CI: 8.36–11.72 months) and 8.79 months for decitabine (95% CI: 7.62–9.96 months), with no statistical significance between them (*p* = 0.386).

Comparative analyses were performed, including studies with the approved regimens of both drugs. Table 3 summarises the response outcomes, OS and 1-year mortality rate for azacitidine and decitabine approved regimens (75 mg/m^2^ for 7 days, and 20mg/m^2^ for 5 days, respectively, available in Supplementary Figures S1–S3). A significantly lower CR was observed for azacitidine (16%, 95% CI: 12%–20%) compared to decitabine (24%, 95% CI: 18%–30%) (*p* < 0.025). However, these differences did not remain significant for the ORR. The 1-year mortality was significantly different between azacitidine (54% mortality, 95% CI: 47%–61%) and decitabine (72% mortality, 95% CI: 67%–76%) (*p* < 0.001). The median OS was 10.83 months (95% CI: 9.07–12.59 months) for azacitidine and 8.46 months (95% CI: 7.00–9.93 months) for decitabine (*p* = 0.138).

No significant differences were found comparing the 5-day and 7-day regimens of azacitidine regarding the ORR (36%, 95% CI: 13–60% versus 41%, 95% CI: 32–50%, *p* = 0.727). However, the 1-year mortality rate was higher in patients following the 5-day regimen (72%, 95% CI: 61–82%) compared to the 7-day regimen (54%, 95% CI: 47–61%) (*p* = 0.008). Additionally, the median OS was lower when the drug was administered for 5 days (6.28 months, 95% CI: 4.23 months–8.32 months) versus 7 days (10.83 months, 95% CI: 9.07 months–12.59 months) (*p* = 0.002).

Again, no significant differences were found when the 5-day (approved) and 10-day regimens of decitabine were compared regarding the ORR (46%, 95% CI: 42–50% vs. 37%, 95% CI 29–46%, *p* = 0.088). We found no significant differences in mortality and the OS either.

**Figure 5:**
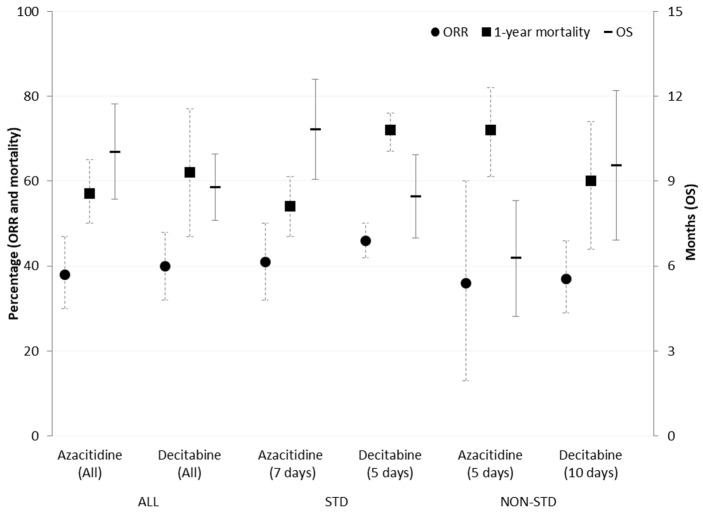
Summary of results. Abbreviation: ORR, overall response rate; OS, overall survival; STD, standard; Non-std, non-standard.

A summary of the results is briefly depicted in Figure 5.

### 3.4. Risk of Bias

**Figure 6:**
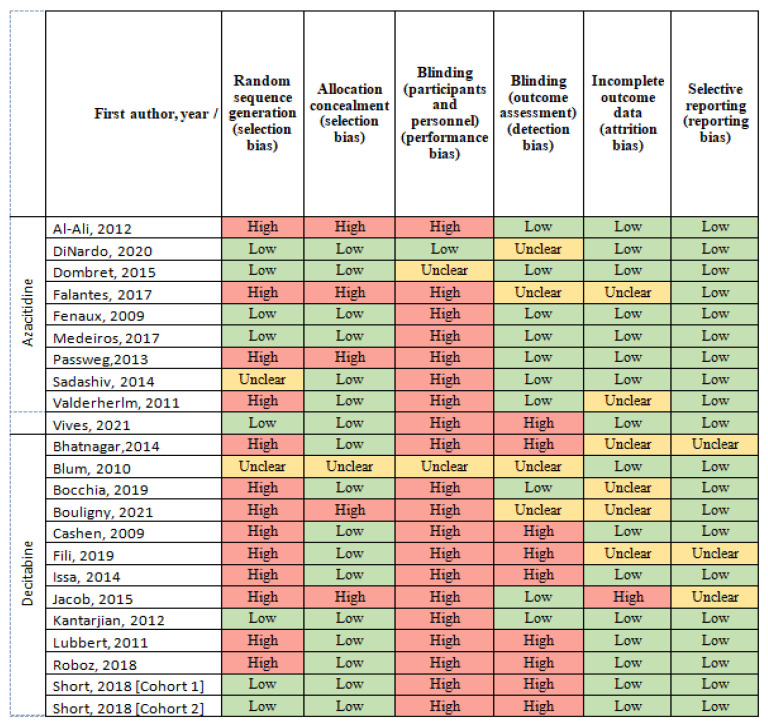
Risk of bias.

Overall, the risk of bias ranged from unclear to high. The risk of bias is shown in Figure 6.

## 4. Discussion

In this systematic review and meta-analysis, we analysed the effectiveness of 23 cohorts including 2743 AML patients treated with upfront azacitidine or decitabine monotherapy in the context of randomised controlled trials and retrospective studies.

As far as we know, this is the first meta-analysis comparing all published pooled azacitidine and decitabine studies according to dose regimens for each drug. Although we found no significant differences in survival between azacitidine and decitabine therapy, the results of this meta-analysis support the effectiveness of the standard dose schemes of these drugs over other dosages.

The efficacy of both HMAs has been compared in two recent studies: (1) a population-based study using the Surveillance, Epidemiology, and End Results (SEER)-Medicare database [42] and (2) a network meta-analysis including 538 patients treated with HMAs from three randomised controlled trials (two for azacitidine and one for decitabine) [43]. Our review includes all randomised clinical trials and retrospective studies (10 for azacitidine and 13 for decitabine, including 2743 patients), and evaluates different dose schedules, which provides a more complete picture of the efficacy of HMAs.

The scarcity of well-controlled studies performed in the older AML population underscores the need for well-conducted meta-analyses. Given that most of the available studies report on small patient samples, it seems mandatory to group all available cohorts. The main limitation of this study was the inclusion of retrospective single-centre studies, increasing the risk of selection bias and heterogeneity. Moreover, data were included from all reported azacitidine and decitabine treatment regimens, but the comparison between the approved regimens and others could have addressed this issue. Accordingly, the results of this meta-analysis may be useful to estimate the effectiveness of HMA monotherapy but may not necessarily apply to new combinations. However, since HMAs are being used as the backbone for novel combinations, the HMA dose schedule that could be more appropriate to combine (e.g., the less toxic and/or more efficacious HMA according to the partner agent) could be selected.

The current analysis confirmed moderate disease control with HMA monotherapy (pooled CR rate 21% (95% CI: 17–25%)). A prior phase 3 trial of decitabine showed a CR rate of 15.7% and a CCR of 25.6%, whereas in another phase 3 trial, azacitidine achieved a CR of 19.5% and a CCR of 27.8% [16,17]. The ORR of these phase 3 trials for azacitidine and decitabine was 31.1% and 45.9%, respectively, which was similar to that observed in this study (38% for azacitidine and 40% for decitabine). Of note, when standard dose schedules of both agents were compared, the ORR found in this meta-analysis, which included retrospective and real-life studies, was identical to that of the phase 3 trials (41% for azacitidine and 46% for decitabine). This difference was not statistically significant.

The presence of p53 mutation leads to extremely poor prognosis [44,45]. Bories et al. described a worse OS in patients with any p53 mutation and treated with azacitidine but found no association for response [46]. Regarding the use of decitabine in p53-mutated AML, Welch et al. found a higher response rate in patients with p53 mutations compared to wild-type patients after 10-day treatment (100% and 41%, respectively) [47]. Aldoss et al. described a comparable response rate between patients treated with venetoclax in combination with either a 5- or a 10-day regimen of decitabine [48]. As these results are not profoundly conclusive, the systematic classification of patients with p53 mutations will merit further investigation in conventional therapy and in the emerging field of p53-pathway-targeted therapies [46].

Although higher response rates have been initially reported with a 10-day schedule of decitabine [18], a recent phase II study did not confirm this superiority in comparison to the 5-day schedule [19]. The results of this study confirm similar response rates, mortality and OS between both decitabine schedules, supporting the use of the standard dose. In contrast, there are no previously reported studies comparing different dose schedules for azacitidine. Again, no statistically significant differences were found in the ORR between the 5 days vs. the standard 7-day azacitidine regimen, which might support the off-label use of this more convenient regimen over the standard 7 days. However, based on the significantly higher 1-year mortality and lower OS observed in this study with the 5-day schedule of azacitidine, it is suggested that azacitidine administered on a schedule other than the indicated 7-day regimen may lead to inferior outcomes.

The OS of azacitidine-treated patients in this study (median, 10.04 months) was similar to that reported in the intention-to-treat population from an AZA-AML-001 trial (median, 10.4 months) [17], while patients on decitabine achieved a median OS of 8.79 months, slightly higher than that observed in the DACO-016 trial (7.7 months) [16]. A recent population-based survey showed similar survival (median OS, 7.1 vs. 8.2 months) for older AML patients treated with azacitidine or decitabine [42]. However, decitabine-treated patients were younger, had fewer comorbid conditions and were more likely to receive the standard dosing schedule than azacitidine-treated patients [42]. Indeed, after adjustment for all these factors in a multivariable analysis, the differences in survival remained statistically significant [42]. A network-meta-analysis including three randomised clinical trials showed that azacitidine improves the OS using SUCRA analysis compared to decitabine, but the authors concluded that the superiority of either agent could not be confirmed, and head-to-head clinical trials are still needed [43]. When the analysis is performed including only studies that used azacitidine and decitabine at standard doses, the median OS was again similar (10.83 months vs. 8.46 months). The results of this meta-analysis support the use of HMAs in the real-world setting and show that single-centre and retrospective cohorts exhibit a median OS that encompass the estimates in the phase 3 trials.

Currently, the combination of HMAs and venetoclax has become a standard of care for AML patients unfit for intensive chemotherapy. DiNardo et al. showed a 73% CR/CRi rate for the combination of venetoclax with HMAs, with a median OS of 17.5 months [49]. Comparing HMAs, they found that response rates and the OS were similar between the venetoclax + azacitidine and venetoclax + decitabine cohorts [49].

A recent study of newly diagnosed AML patients by Pollyea et al. showed a CR/CRi rate of 71% in patients treated with venetoclax and azacitidine and of 74% in patients treated with venetoclax and decitabine [50], showing a higher CR/CRi duration for venetoclax + azacitidine (21.9 months) compared to venetoclax + decitabine (15.0 months) [50]. However, the OS did not differ between combination regimens (16.4 months for venetoclax + azaciditine and 16.2 months for venetoclax + decitabine) [50]. All these findings are in concordance with our results regarding the similarity of treatment outcomes using either azaciditine or decitabine in combination with venetoclax.

## 5. Conclusions

Despite remarkable heterogeneity between the different studies, we found no significant differences regarding the 1-year mortality and OS for azacitidine and decitabine (roughly 9 months) in AML patients. Furthermore, this study shows that there are no significant differences in the efficacy of 5-day versus 10-day schedules of decitabine. However, patients treated with a shortened 5-day azacitidine scheme showed worsening outcomes compared to the standard 7-day regimen. Hopefully, the results of this meta-analysis, exploring single HMA regimens, might be helpful for the design of HMA-based combination schedules to be tested in future trials.

## Figures and Tables

**Table 1:** Patient characteristics of studies included in the meta-analysis.

Variable	Study (First Author, Year)	Study Design	Intervention	Comparison	Patients (N)	Age (Years)	Male (%)	ECOG 0/1, 2, ≥3	AML Type, DN, S (%)	Cytogenetics F, I, A (%)	NPM1 (M, W, NA) (%)	FLT3 (M, W, NA) (%)	BM Blasts (%)	WBC Count (10^9^/L)
Azacitidine	Al-Ali, 2012 [20]	Phase I/II	75 mg/m^2^/day SC for 5 days every 4 weeks	NA	20	78 (64–84)	55		40, 60	75, 25	15, 75, 10	15, 75, 10	44 (10–90)	3.4 (0.8–187.3)
	DiNardo, 2020 [26]	Phase III	75 mg/m^2^/day SC for 7 days every 4 weeks	Aza + Ven	145	76 (60–90)	60	56.44	76, 24	0, 61, 39	12, 47, 41	15, 59, 26		
	Dombret, 2015 [17]	Phase III	75 mg/m^2^/day SC for 7 days every 4 weeks	Conventional care regimen	241	75 (64–91)	57.7	77.2; 22.8	80, 20	0, 65, 35			70 (2, 100)	3.1 (0–33)
	Falantes, 2017 [27]	Retrospective	75 mg/m^2^/day SC for 7 days every 4 weeks	NA	710	75 (60–93)	63.1	65; 27.7	44, 56	1.5; 53.1; 31.3			38 (1–98)	2.9 (0.1–190)
	Fenaux, 2009 [28]	Phase III	75 mg/m^2^/day SC for 7 days every 4 weeks	Conventional care regimen	55	70 (52–80)	67.3	92.7; 7.3		34.5; 69.1; 25.5			23 (20–34)	
	Medeiros, 2017 [29]	Phase II	75 mg/m^2^/day SC for 7 days every 4 weeks	Lenalidomide	34	75 (66–85)	55.9	79.4; 17.6	85.3; 14.7	50			34 (14–70)	
	Passweg, 2013 [21]	Phase III	100 mg/m^2^/day SC for 5 days every 4 weeks	NA	45	74 (55–86)	60							
	Sadashiv, 2014 [30]	Phase II	100 mg/m^2^/day SC for 5 days every 4 weeks	NA	15	74 (64–82)	60			60; 33, 3			44 (29–92)	2.9 (1.2–43.3)
	van der Herlm, 2011 [31]	Retrospective	75 mg/m^2^/day SC for 7 days every 4 weeks	NA	31	71 (40–84)	74			0; 68; 32				
	Vives, 2021 [32]	Phase III	75 mg/m^2^/day subcutaneously for 7 days every 4 weeks	FLUGA	142	74 (65–90)	60	75.25	79; 21				52 (10–99)	4.5 (0.6–235.5)
Decitabine	Bhatnagar, 2014 [33]	Retrospective	20 mg/m^2^/day IV for 10 days every 4 weeks	NA	45	74 (52–87)	56	58; 42		2; 42; 53	4; 49; 47	13; 62; 24	49 (18–96)	7.7 (0.8–117.2)
	Blum, 2010 [18]	Phase II	20 mg/m^2^/day IV for 10 days every 4 weeks	NA	53	74 (60–85)	64						52 (20–92)	2.7 (0.4–150.0)
	Bocchia, 2019 [34]	Retrospective	20 mg/m^2^/day IV for 5 days every 4 weeks	NA	306	75 (65–90)			59.5; 40.5	3.6; 50.3; 30.4				4.5 (1.8–17.1)
	Bouligny, 2021 [35]	Prospective	20 mg/m^2^/day IV for 10 days every 4 weeks	NA	72	74 (44–88)	68			4.2; 44.4; 51.4				3.0
	Cashen, 2009 [36]	Phase II	20 mg/m^2^/day IV for 5 days every 4 weeks	NA	55	74 (61–87)	50	82; 18	55; 42	53; 45			50 (0–99)	2.7 (1–111)
	Fili, 2019 [37]	Retrospective	20 mg/m^2^/day IV for 5 days every 4 weeks	NA	75	74	53.3	88; 12	56, 44			11.4; 88.6		3.4 (0.8–25.5)
	Issa, 2004 [38]	Phase II	20 mg/m^2^/day IV for 5 days every 4 weeks	Decitabine + valproic acid	62	70 (38–83)								
	Jacob, 2015 [39]	Prospective	20 mg/m^2^/day IV for 5 days every 4 weeks	LDC	15	65 (60–80)	80	53.3; 46.7	87, 13				40	
	Kantarjian, 2012 [16]	Phase III	20 mg/m^2^/day IV for 5 days every 4 weeks	TC	242	73.0 (64.0–89.0)	56.6	76; 24	64; 36	63.1; 36.1				3.10 (0.3–127.0)
	Lubbert, 2011 [40]	Phase II	135 mg/m^2^ total dose infused IV over 72 h every 6 weeks	NA	227	72 (56–86)	61.2	77; 22.1; 0.9			7.3; 92.6;	7.9; 89	56 (10–100)	4.4 (0.5–241)
	Roboz, 2018 [41]	Phase II	20 mg/m^2^/day IV for 10 days every 4 weeks	Decitabine + bortezomib	82	72.4 (60.7–92.3)	62.2	76.8; 19.5; 3.7	69.5; 30.5	28.2; 26.8; 45.1				13.3 (0.4–212.7)
	Short, 2018 (cohort 1) [19]	Phase II	20 mg/m^2^/day IV for 5 days every 4 weeks	NA	28	77 (70–80)		64; 36	54; 46		4; 96	8; 92	40 (29–68)	2.0 (1.5–3.9)
	Short, 2018 (cohort 2) [19]	Phase II	20 mg/m^2^/day IV for 10 days every 4 weeks	NA	43	78 (69–82)		70; 30	58; 42		19; 81	5; 95	46 (25–64)	3.2 (1.9–10.6)

Abbreviations: NA, not applicable; ECOG, Eastern Cooperative Oncology Group; AML, acute myeloid leukaemia; DN, de novo; S, secondary; F, favourable; I, intermediate; A, adverse; M, mutated; W, wild type; BM, bone marrow; WBC, white blood cell.

**Table 2:** Efficacy outcomes of the studies included in the meta-analysis.

Variable	Study (First Author, Year)	CR (%)	CCR (%)	PR (%)	ORR (%)	Median OS (Months)	30-Day Mortality (%)	60-Day Mortality (%)	Mortality (%)
Azacitidine	Al-Ali, 2012 [20]	10		15	50	7.7 (0.2–14.8)			61
	DiNardo, 2020 [26]	17.9	28.3			9.6 (7.4–12.7)	6		
	Dombret, 2015 [17]	19.5	27.8	1,2	48.5	12,1 (9.2–14.2)	6.6	16.2	49.3
	Falantes, 2017 [27]				35.5	9,0 (8.8–11)			60.8
	Fenaux, 2009 [28]	18				24,5 (14.6–38)			
	Medeiros, 2017 [29]	17.6	41.2				5.9	8.8	48
	Passweg, 2013 [21]		17.8	0	17.8	6 (3.4–7.8)		18	75.6
	Sadashiv, 2014 [30]	20		13	47	11.8 (0.4–30.3)			
	van der Herlm, 2011 [31]	16	23	3	26	13.0 (9.8–16.2)			
	Vives, 2021 [32]	9	13	28	50	9.8 (5.6–14)			53
Decitabine	Bhatnagar, 2014 [33]	31	42			9.0 (3.9–14.2)	4		61
	Blum, 2010 [18]	47	64			13.7(9–18)		15	
	Bocchia, 2019 [34]	23.2	14.7	10.5	48.4	10.0 (7.9–11.9)			77.8
	Bouligny, 2021 [35]	16.7	36.1		36.1		5.6	19.4	
	Cashen, 2009 [36]	24	26			7.7 (5.7–11.6)	7		
	Fili, 2019 [37]	31		11	42	12.7 (0.1–22.5)			37
	Issa, 2004 [38]	33				9.6 (1–59.0)			
	Jacob, 2015 [39]					5.5 (0.5–13)			
	Kantarjian, 2012 [16]	15.7	25.6	2.5	43.8	7.7 (6.2–9.2)	9	19.7	
	Lubbert, 2011 [40]	13.21		12.77	25.98	5.5 (1–36.0)			72
	Roboz, 2018 [41]		39			9.3 (5.8–12.2)			
	Short, 2018 (cohort 1) [19]	29	4	0	43	5.5 (2.1–11.7)	4	21	
	Short, 2018 (cohort 2) [19]	30	5	10.5	40	6.0 (1.9–11.7)	9	25	

Abbreviations: CR, complete remission; CCR, composite complete remission rate; PR, partial response; ORR, overall response rate; OS, overall survival.

**Table 3:** Summary of response outcomes, overall survival and mortality during azacitidine (75 mg/m^2^, 7d) and decitabine treatment (20mg/m^2^, 5d) in monotherapy.

Drug	CR %, 95% CI	ORR %, 95% CI	1-Year Mortality %, 95% CI	OS (Months) 95% CI
Azacitidine	16% (12–20)	41% (32–50)	54% (47–61)	10.83 (9.07–12.59)
(75 mg/m^2^, 7d)	I^2^ = 54.41%	I^2^ = 85.10%	I^2^ = 75.71%	
Decitabine (20mg/m^2^, 5d)	24% (18–30)	46% (42–50)	72% (67–76)	8.46 (7.00–9.93)
	I^2^ = 63.54%	I^2^ = 0%		
*p*-value	0.025	0.327	<0.001	0.138

Abbreviation: CR, complete remission; ORR, overall response rate; OS, overall survival. I^2^ coefficient >50% suggests substantial heterogeneity.

## Data Availability

The data that support the findings of this study are available from the corresponding author upon reasonable request.

## Supplementary Materials

The following are available online at www.mdpi.com/article/10.3390/cancers13225677/s1, Figure S1: Overall response rate (ORR) analysis of azacitidine approved regimen (75mg/m^2^ for 7 days) versus decitabine approved regimen (20mg/m^2^ for 5 days), Figure S2: 1-year mortality analysis of azacitidine approved regimen (75mg/m^2^ for 7 days) versus decitabine approved regimen (20mg/m^2^ for 5 days), Figure S3: Overall survival (OS) analysis of azacitidine approved regimen (75mg/m^2^ for 7 days) versus decitabine approved regimen (20mg/m^2^ for 5 days), Table S1: Complete search strategy in the different reviewed databases.
