# Supplementary material for: Use of Azacitidine or Decitabine for the Up-Front Setting in Acute Myeloid Leukaemia: A Systematic Review and Meta-Analysis
Source: Cancers (Basel). 2021 Nov 12;13(22):5677. doi: 10.3390/cancers13225677 (PMC8616518; doi:10.3390/cancers13225677)
Supplement: Supplementary file 1 [file cancers-13-05677-s001.zip › cancers-1451457-supplementary.pdf]

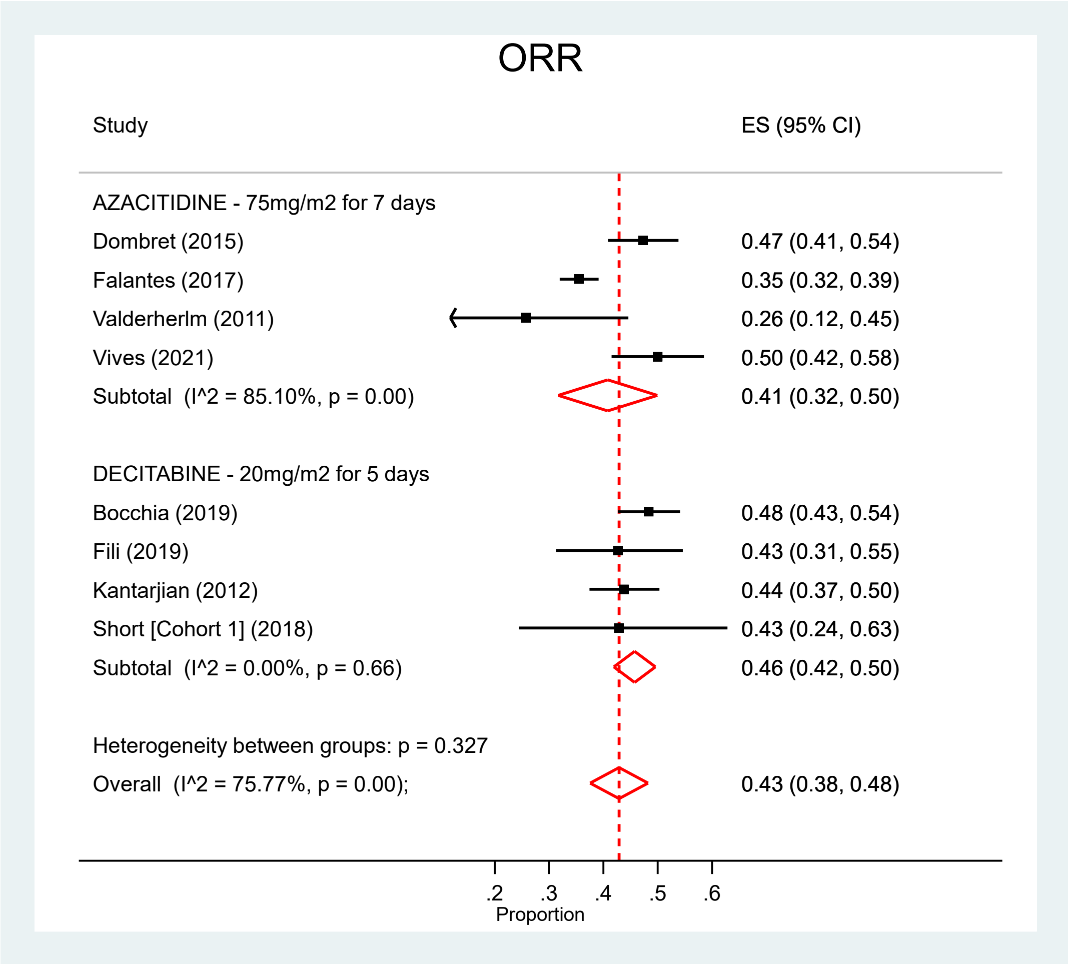

Supplementary figure S1. Overall response rate (ORR) analysis of azacitidine approved regimen (75mg/m^2 for 7 days) versus decitabine approved regimen (20mg/m^2 for 5 days).

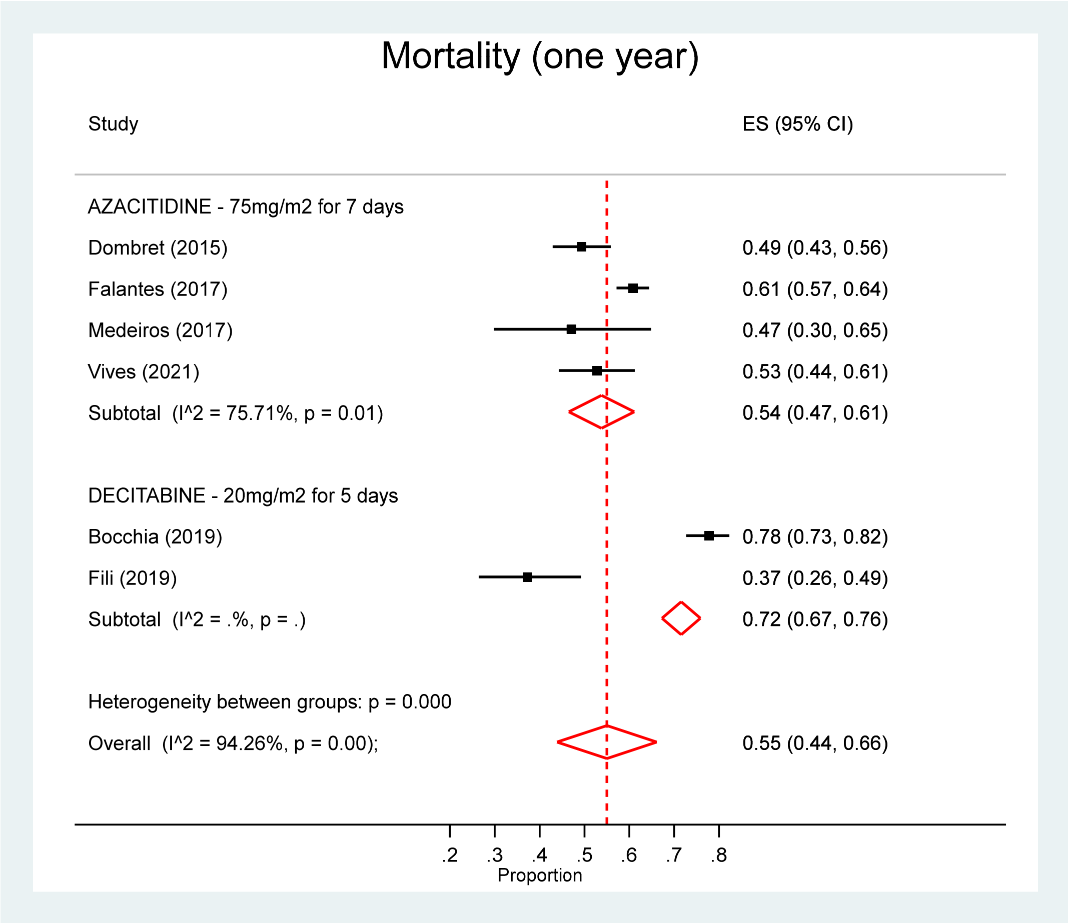

Supplementary figure S2. 1-year mortality analysis of azacitidine approved regimen (75mg/m^2 for 7 days) versus decitabine approved regimen (20mg/m^2 for 5 days).

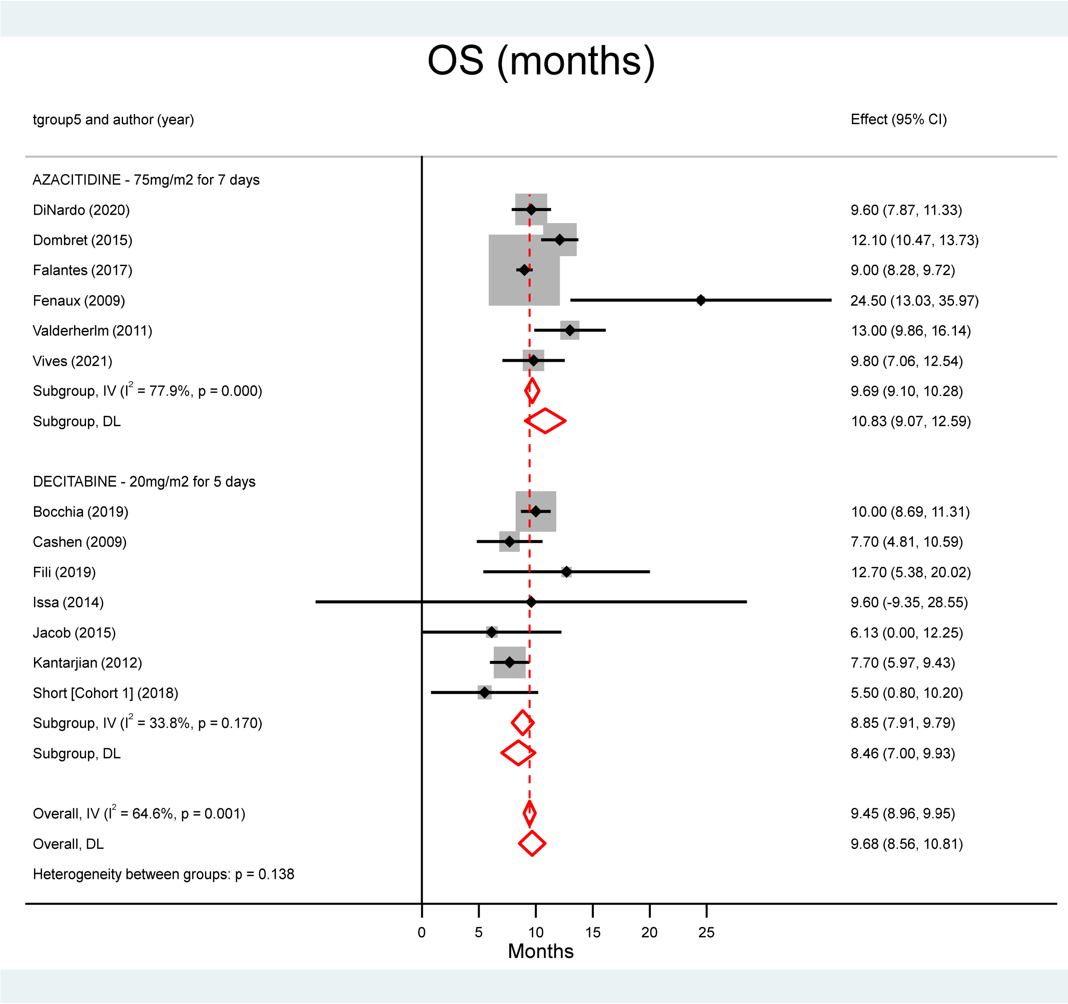

Supplementary figure S3. Overall survival (OS) analysis of azacitidine approved regimen (75mg/m^2 for 7 days) versus decitabine approved regimen (20mg/m^2 for 5 days).

Supplementary table S1. Complete search strategy in the different reviewed databases.

AGENT	DATABASE	SEARCH STRATEGY
**AZACITIDINE**	Medline (searched through Pubmed)	((((((acute myeloid[Title/Abstract] AND leukemia[Title/Abstract]) OR ("leukaemia"[Title/Abstract] OR "leukemia"[MeSH Terms] OR "leukemia"[Title/Abstract])) AND older[Title/Abstract]) OR ("aged"[MeSH Terms] OR "aged"[All Fields] OR "elderly"[Title/Abstract])) OR unfit[Title/Abstract]) AND ("azacitidine"[MeSH Terms] OR "azacitidine"[Title/Abstract])) AND ("clinical trials as topic"[MeSH Terms] OR ("clinical"[All Fields] AND "trials"[All Fields] AND "topic"[All Fields]) OR "clinical trials as topic"[All Fields] OR "trial"[All Fields])
	Cochrane Central Register of Controlled Trials	"acute myeloid leukemia" and "azacitidine"
	EU Clinical Trials Register	acute myeloid leukemia AND azacitidine; trials with results
	ClinicalTrials.gov	Condition: acute myeloid leukemia<br>Intervention: azacitidine<br>Other terms: older/elderly/unfit<br>Studies with results
**DECITABINE**	Medline (searched through Pubmed)	((((((acute myeloid[Title/Abstract] AND leukemia[Title/Abstract]) OR ("leukaemia"[Title/Abstract] OR "leukemia"[MeSH Terms] OR "leukemia"[Title/Abstract])) AND older[Title/Abstract]) OR ("aged"[MeSH Terms] OR "aged"[All Fields] OR "elderly"[Title/Abstract])) OR unfit[Title/Abstract]) AND ("decitabine"[MeSH Terms] OR "decitabine"[Title/Abstract])) AND ("clinical trials as topic"[MeSH Terms] OR ("clinical"[All Fields] AND "trials"[All Fields] AND "topic"[All Fields]) OR "clinical trials as topic"[All Fields] OR "trial"[All Fields])
	Cochrane Central Register of Controlled Trials	"acute myeloid leukemia" and "decitabine"
	EU Clinical Trials Register	acute myeloid leukemia AND decitabine; trials with results
	ClinicalTrials.gov	Condition: acute myeloid leukemia<br>Intervention: decitabine<br>Other terms: older/elderly/unfit<br>Studies with results
